# 

**Published:** 1904-07

**Authors:** 


### ATLANTA JOURNAL-RECORD OF MEDICINE.

Successor to Atlanta Medical and Surgical Journal, Established 1855,
and Southern Medical Record, Established 1870.

VOL. VI. ATLANTA, GA., JULY, 1904. No. 4.

No physician can afford to be indifferent regarding the accurate filling of his prescriptions.

